# Successful Treatment of Multifocal Histiocytic Sarcoma Occurring after Renal Transplantation with Cladribine, High-Dose Cytarabine, G-CSF, and Mitoxantrone (CLAG-M) Followed by Allogeneic Hematopoietic Stem Cell Transplantation

**DOI:** 10.1155/2015/728260

**Published:** 2015-06-08

**Authors:** Julia Tomlin, Ryan K. Orosco, Sarah Boles, Ann Tipps, Huan-You Wang, Jacob Husseman, Matthew Wieduwilt

**Affiliations:** ^1^Division of Otolaryngology-Head & Neck Surgery, Department of Surgery, University of California San Diego, San Diego, CA, USA; ^2^Division of Hematology-Oncology, Department of Medicine, University of California San Diego, San Diego, CA, USA; ^3^Department of Pathology, University of California San Diego, San Diego, CA, USA; ^4^Blood and Marrow Transplantation Program, Moores Cancer Center, 3855 Health Sciences Drive, No. 0960, La Jolla, CA 92093-0960, USA

## Abstract

Histiocytic sarcoma (HS) is a rare, aggressive malignancy. Lesions previously called HS were typically non-Hodgkin lymphomas, not HS. As such, chemotherapy directed at lymphoid neoplasms was frequently successful, but it is unclear if these regimens are ideal for HS. We present a 33-year-old African gentleman who underwent sequential renal transplants for glomerulonephritis. He subsequently developed HS of the upper airway and multiple cutaneous sites. The patient received cyclophosphamide, doxorubicin, vincristine, and prednisone (CHOP) followed by salvage ifosfamide, carboplatin, and etoposide (ICE) but had continuous progression of cutaneous involvement. Cladribine, high-dose cytarabine, G-CSF, and mitoxantrone (CLAG-M) yielded a partial response with near resolution of disease. Ultimately, the patient achieved a complete remission after myeloablative allogeneic hematopoietic stem cell transplant. HS occurring after solid organ transplant raises the possibility of HS as a potential posttransplant malignancy. The use of CLAG-M has not been reported in HS. In this case, histiocyte-directed chemotherapy with CLAG-M was superior to lymphoma-directed regimens.

## 1. Introduction

Histiocytic sarcoma [1] is a rare neoplasm defined in the WHO classification of histiocytic and dendritic cell neoplasms comprised of malignant cells showing morphological and immunophenotypic characteristics of mature histiocytes [2]. It is an extremely rare disease of adulthood, accounting for only a small percentage of all lymphomatoid neoplasms. HS presents at a mean age of 46 with no apparent gender or hereditary predictors [3]. Though sometimes occurring in conjunction with non-Hodgkin's lymphoma and germ cell tumors, no precursor lesion or etiologic agent has yet been identified. Most cases of HS follow an aggressive clinical course, with most patients dying of progressive disease within one year of diagnosis [4].

The term “histiocytic lymphoma” has evolved considerably over the years and was previously used interchangeably with HS. However, retrospective studies have shown that almost all lesions previously called histiocytic lymphoma were, in fact, B-cell or T-cell immunoblastic or, more commonly, anaplastic large cell lymphomas, not true histiocytic lesions [3, 5, 6]. In the current literature, the term “true histiocytic lymphoma” is now used synonymously with histiocytic sarcoma.

HS with head and neck manifestations is particularly rare, and few cases have been presented in the literature. De Vos et al. described a case presenting as a neck mass and hypothyroidism in a 64-year-old woman [7]. Similarly, Yu and Yang reported primary HS in a 69-year-old man with bilateral nodular enlargement of the thyroid gland [8]. In 2007, Alexiev et al. presented the first case of HS with predominant spindle cell component in a 41-year-old man with preauricular swelling, headache, jaw pain, and trismus [9]. Akiba et al. reported HS of the parotid gland in a 53-year-old woman with a painful preauricular mass [1]. Several cases of HS have been detailed in the brain and central nervous system [10–17].

Unlike localized disease which can be effectively managed with surgery or radiation therapy, multifocal HS follows an aggressive course, with most patients dying of disease within one year of diagnosis [4]. Given the frequent misdiagnosis of non-Hodgkin lymphomas as HS, lymphoma-directed chemotherapy regimens such as CHOP appeared successful in HS although it is unclear if true HS responds well to these regimens. Given the poor response of true HS to lymphoma-directed therapy and its dismal prognosis, alternative therapeutic approaches are needed. We describe a case of disseminated HS of the upper airway and skin occurring in a man who had undergone two renal transplants.

## 2. Case Description

This 33-year-old gentleman was born and raised in central Africa and his past medical history was significant for two renal transplants for glomerulonephritis. He was maintained on immunosuppression therapy with mycophenolate, tacrolimus, and prednisone. He presented to his nephrologist complaining of several months of throat pain. He was treated with esomeprazole for a presumed diagnosis of gastroesophageal reflux. This provided temporary relief, but, over the ensuing three months, symptoms progressed to include dysphagia for pills and solids and a progressive cough with increased mucus secretions.

**Figure 1:**
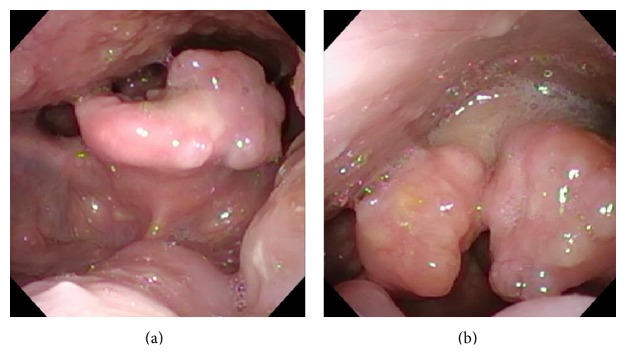
Color photographs taken during flexible indirect laryngoscopy. The vallecula is uninvolved, and an irregular mass is seen on the laryngeal surface of the epiglottis (a). Irregular, submucosal growths are seen on bilateral arytenoids, with pooling of secretions in the postcricoid region (b).

**Figure 2:**
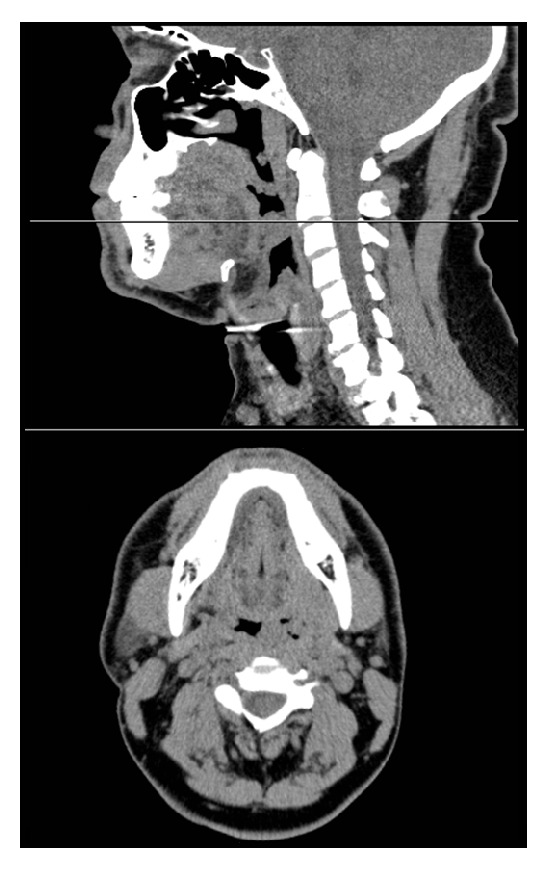
Noncontrast computed tomography. Sagittal and axial slices demonstrate a narrowed airway and thickening of the epiglottis (cursor shown for level) and subcentimeter cervical lymph nodes.

Worsening symptoms prompted his presentation to the emergency department where he was referred for otolaryngology evaluation. Flexible indirect laryngoscopy revealed multiple lesions along Waldeyer's ring and the supraglottic region. There was an irregularity in the inferior aspect of left tonsil and an exophytic irregular mass involving the laryngeal surface of the epiglottis. Similar lesions were present on the arytenoids (Figure 1). All lesions appeared to be submucosal in nature with smooth surfaces and no visible ulceration. A CT scan of the neck showed multiple polypoid masses involving the supraglottis with some airway narrowing, lobulated margins of the palatine tonsils, and scattered subcentimeter cervical lymph nodes (Figure 2).

**Figure 3:**
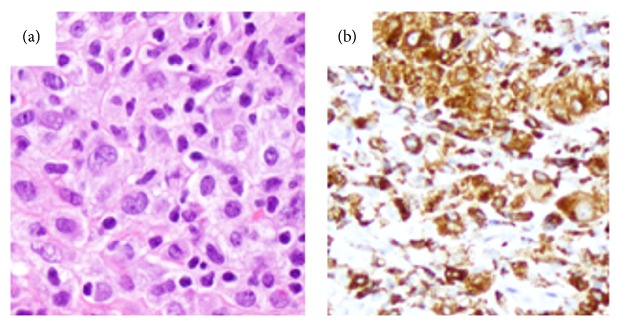
(a) Microphotograph of the histiocytic sarcoma cells. At high magnification, the neoplastic cells from the initial oropharynx biopsy are medium sized to large cells with irregular nuclear contours, delicate nuclear membrane, and abundant pale cytoplasm, consistent with atypical histiocytes (H&E, original magnification 400x). (b) Immunohistochemical features of the histiocytic sarcoma cells. The sarcoma cells are positive for CD68 (b, original magnification 200x), lysozyme (not shown), and CD45 (not shown). Approximately 10% of the sarcoma cells are positive for Ki-67 (not shown).

The patient underwent microscope-assisted direct laryngoscopy with multiple directed biopsies of the suspicious lesions. Histopathology revealed a collection of atypical histiocytes staining positive by immunohistochemistry for CD45, CD45-RO, CD68, LCA, Pan-LCA, S-100 (patchy), and lysozyme (Figure 3). Malignant cells were negative for CD1a, CD21, CD30, CD3, CD20, and ALK with near absence of MPO staining. LMP1 immunohistochemistry and EBER* in situ *hybridization were negative. The final diagnosis was histiocytic sarcoma.

**Figure 4:**
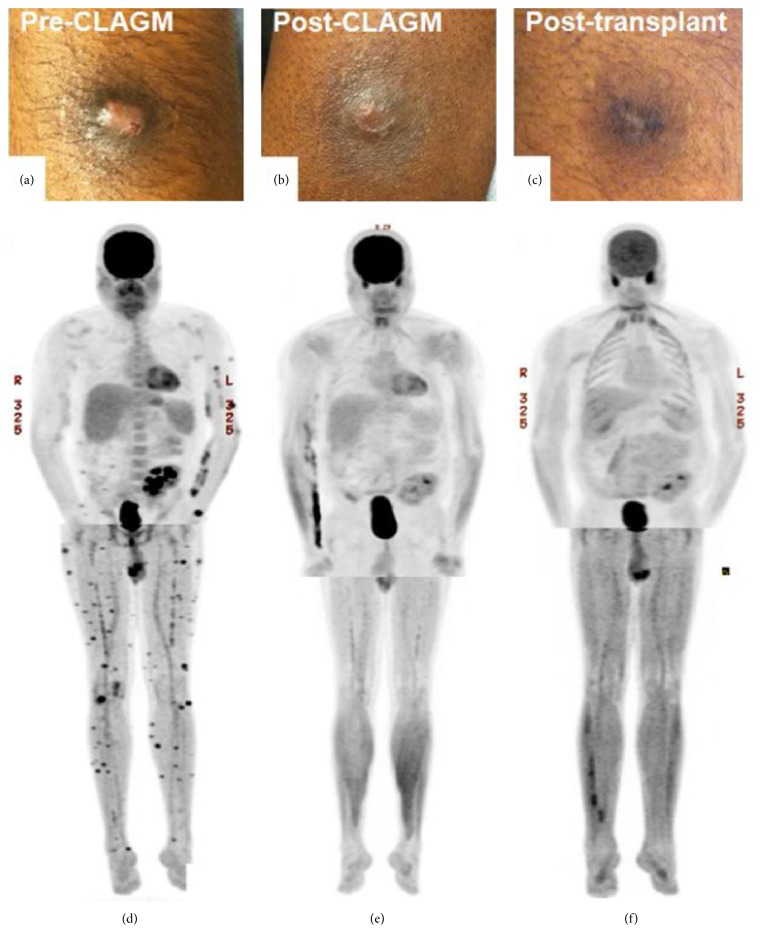
(a, b, and c) Photographs of a pre-CLAG-M, post-CLAG-M, and postallogeneic hematopoietic cell transplant skin lesion. (d, e, and f) Maximum intensity projection from positron emission tomography (PET) of upper body and lower extremity. Images correspond with timing of skin lesions photographed in (a)–(c). (d) After CHOP (cyclophosphamide, doxorubicin, vincristine, and prednisone) and salvage ifosfamide, carboplatin, etoposide (ICE) there was progression of skin disease with innumerable cutaneous foci on PET. (e) Following CLAG-M therapy, near resolution of skin lesions. PET demonstrated improvement in cutaneous foci consistent with response to therapy. (f) Five months following allogeneic hematopoietic cell transplant there is no evidence of metabolically active tumor.

A PET/CT demonstrated hypermetabolic activity of the palatine tonsils, epiglottis, and bilateral aryepiglottic folds. There were no positive neck nodes, but there was hypermetabolic activity within two subcutaneous nodules overlying the right lateral hip and left upper thigh (Figure 4). Repeat endoscopy revealed additional abnormal tissue in the nasopharynx and along the left inferior turbinate, along with the previously noted supraglottic disease. In order to more fully characterize this rare neoplasm, nasal endoscopy, repeat direct laryngoscopy, and bilateral tonsillectomy were performed. The left inferior turbinate and bilateral nasopharynx demonstrated the same histiocytic neoplasm seen previously but the tonsillectomy specimens showed no evidence of malignancy. A bone marrow biopsy was normal.

The patient received cyclophosphamide, doxorubicin, vincristine, and prednisone (CHOP). After three cycles, his symptoms resolved and upper-airway endoscopy converted to normal. PET/CT showed no abnormal FDG uptake in the neck but increased areas of subcutaneous hypermetabolic activity. He developed hyperpigmented nodules on the arms and legs that biopsies proved to be histiocytic sarcoma. He received salvage ifosfamide, carboplatin, and etoposide (ICE) with further progression of skin disease. Cladribine, high-dose cytarabine, G-CSF, and mitoxantrone (CLAG-M) yielded a partial response with near resolution of skin lesions on exam and PET/CT. The dosing schedule for CLAG-M is listed in Table 1. He received 2 cycles of weekly vinblastine then myeloablative cyclophosphamide/TBI allogeneic hematopoietic cell transplant from a matched sibling donor. Skin nodules resolved over the ensuing 2 months. Sixty days after transplant, a PET/CT was without evidence of metabolically active tumor (Figure 4). The patient died 9 months after transplant from pneumonia. Autopsy revealed no evidence of HS.

## 3. Discussion

Sarcomas of the head and neck are extremely rare entities, accounting for less than 1% of all malignancies in the area [18]. Histiocytic sarcoma is an aggressive, high-grade tumor that is typically identified at an advanced stage. In addition to having diagnostic complexity, there are no guidelines or established standard of care for the treatment of HS. Optimal treatment for disseminated HS has not been defined but, given in large part the historical misdiagnosis of non-Hodgkin lymphomas as HS, it is typically approached as a lymphoma with systemic chemotherapy.

Prior studies involving extranodal HS have employed chemotherapy, frequently CHOP or CHOP-like regimens, in initial treatment plus or minus adjuvant radiation, with ICE therapy as a salvage [1, 9]. Localized disease has a fairly good prognosis with local therapy such as XRT or surgical excision. One report of 14 cases illustrates the case of a nasal cavity HS that responded well to wide excision and radiation [3]. A second report of 5 cases reported 1 case of a localized palatal disease successfully treated with surgery alone. The four other cases died within 15 months of diagnosis from progressive histiocytic sarcoma [19]. HS of the CNS has been treated with a variety of modalities in varying combinations. Perhaps the most common approach has begun with partial resection of the tumor followed by adjuvant radiation and chemotherapy [16]. Other authors report employing radiotherapy alone when the tumor site was not conducive to excision, either using three-dimensional conformal radiation therapy and/or whole brain external beam radiation. Still others have employed repeat resection or chemotherapy alone, though these treatment regimens are less common in the literature [10, 12, 16]. Regardless of treatment regimen, outcomes have thus far been poor with multifocal disease, with nearly all patients reported to experience local or distant recurrence of disease within months following therapy.

For historical reasons, principally misdiagnosis of non-Hodgkin lymphomas as HS, lymphoma-directed therapy such as CHOP or CHOP-like regimens has been used despite a lack of data for superiority over histiocyte-directed therapies [3]. Cladribine, cytarabine, and vinblastine all have documented activity in Langerhans histiocytosis but have not been reported to be active in histiocytic sarcoma [20–26]. Application of CLAG-M to this patient yielded a partial response that was maintained with vinblastine as a bridge to allogeneic HCT. Outcomes of allogeneic HCT hematopoietic cell transplantation for histiocytic sarcoma have rarely been reported and the optimal conditioning regimen is not known [27]. Myeloablative allogeneic HCT with cyclophosphamide/TBI conditioning in this patient ultimately yielded a complete response possibly due to conditioning alone given the radiation responsiveness of this disease and/or graft-versus-tumor effect.

HS occurring after solid organ transplant has been reported rarely in the literature and raises the possibility of HS as a treatment-related neoplasm, as the patient was on long-standing mycophenolate mofetil, or a posttransplant neoplastic disorder. Kramer et al. reported a case of persistent Epstein-Barr virus infection and reported histiocytic sarcoma with positivity for Epstein-Barr nuclear antigen and EBV DNA occurring 1 year after renal transplantation [28]. Notably, Epstein-Barr virus was not detected in the patient's tumor or serum. Castro et al. reported four cases of HS occurring after treatment for acute lymphoblastic leukemia suggesting that HS can be a treatment-related phenomenon or result from possible transdifferentiation of ALL clone as a subset of histiocytic neoplasms in the study shared either clonal marker or a common gene signature with the original ALL clone [4]. Several case reports have documented clonal genetic relationships between prior non-Hodgkin lymphoma and subsequent HS suggesting transdifferentiation [29–33]. Notably, this patient had no documented PTLD and his HS is presumed to be an isolated malignancy.

Use of CLAG-M has not been previously documented in HS but yielded a partial response after disease progression on CHOP and ICE. Ultimately, the patient achieved a complete remission after allogeneic hematopoietic cell transplant using cyclophosphamide/TBI conditioning durable until his death from bacterial pneumonia. In this case of HS, histiocyte-directed chemotherapy was superior to lymphoma-directed regimens for the treatment of HS. This case supports CLAG-M as a possible treatment regimen for HS and the use of allogeneic HCT as consolidation of response to chemotherapy.

## Figures and Tables

**Table 1:** 

CLAG-M regimen as administered to patient	

Cladribine	5 mg/m^2^/day IV days 2–6
Cytarabine	2,000 mg/m^2^/day IV days 2–6
Mitoxantrone	10 mg/m^2^/day IV days 2–4
G-CSF	480 mcg/day SC days 1–6

Cladribine, high-dose cytarabine, G-CSF, and mitoxantrone (CLAG-M) (IV = intravenous; SC = subcutaneous).
